# Microencapsulation of Juniper and Black Pepper Essential Oil Using the Coacervation Method and Its Properties after Freeze-Drying

**DOI:** 10.3390/foods12234345

**Published:** 2023-12-01

**Authors:** Alicja Napiórkowska, Arkadiusz Szpicer, Iwona Wojtasik-Kalinowska, Maria Dolores Torres Perez, Herminia Dominguez González, Marcin Andrzej Kurek

**Affiliations:** 1 Department of Technique and Food Development, Warsaw University of Life Sciences, 02-787 Warsaw, Poland; alicja_napiorkowska@sggw.edu.pl (A.N.); arkadiusz_szpicer@sggw.edu.pl (A.S.); iwona_wojtasik_kalinowska@sggw.edu.pl (I.W.-K.); 2 CINBIO, Departamento de Ingeniería Química, Campus Ourense, Universidade de Vigo, 32004 Ourense, Spain; matorres@uvigo.es (M.D.T.P.); herminia@uvigo.es (H.D.G.)

**Keywords:** essential oils, complex coacervation, gum Arabic, gelatin

## Abstract

Essential oils are mixtures of chemical compounds that are very susceptible to the effects of the external environment. Hence, more attention has been drawn to their preservation methods. The aim of the study was to test the possibility of using the classical model of complex coacervation for the microencapsulation of essential oils. Black pepper (*Piper nigrum*) and juniper (*Juniperus communis*) essential oils were dissolved in grape seed (GSO) and soybean (SBO) oil to minimize their loss during the process, and formed the core material. Various mixing ratios of polymers (gelatin (G), gum Arabic (GA)) were tested: 1:1; 1:2, and 2:1. The oil content was 10%, and the essential oil content was 1%. The prepared coacervates were lyophilized and then screened to obtain a powder. The following analyses were determined: encapsulation efficiency (EE), Carr index (CI), Hausner ratio (HR), solubility, hygroscopicity, moisture content, and particle size. The highest encapsulation efficiency achieved was within the range of 64.09–59.89%. The mixing ratio G/GA = 2:1 allowed us to obtain powders that were characterized by the lowest solubility (6.55–11.20%). The smallest particle sizes, which did not exceed 6 μm, characterized the powders obtained by mixing G/GA = 1:1. All powder samples were characterized by high cohesiveness and thus poor or very poor flow (CI = 30.58–50.27, HR = 1.45–2.01).

## 1. Introduction

In response to consumer expectations, food producers have begun to focus on the production of high-quality and less processed products. These products also come with the longest shelf life. Moreover, a commitment to environmental stewardship has prompted manufacturers to prioritize the reduction or elimination of synthetic preservatives and stabilizers [1]. This has led to a growing emphasis on plant-based alternatives, such as essential oils, to meet these evolving expectations. This is because essential oils have a strong antimicrobial effect, and their use minimizes the risk of foodborne pathogens acquiring resistance to antibiotics. In addition, essential oils have several health-promoting properties [1,2,3].

The main components of juniper essential oil (*Juniperus communis* L.) are α-pinene, limonene, and myrcene [4,5], while the main ingredients of black pepper essential oil (*Piper nigrum* L.) are α-pinene, sabinene, β-pinene, δ-3-carene, limonene, and β-caryophyllene [6,7,8]. Both plants are known worldwide as spices but also as pharmaceutical raw materials [9,10]. They have strong antioxidant and antimicrobial properties [7,11], but also have anti-inflammatory and antispasmodic properties [5].

The growing interest in essential oils and the escalating demand for natural additives in the food industry have prompted research into their powdered forms as potential food ingredients [12]. Encapsulating essential oils has become imperative as these substances are sensitive to external environmental factors such as light, temperature, pH, and oxygen. However, their challenges extend beyond environmental sensitivity. Their lipophilic nature, resulting in low water solubility, poor bio-accessibility, and limited bioavailability, hinders their widespread application in food products [13,14,15]. The application of microencapsulation techniques has been proven to be essential in safeguarding the active ingredients of essential oils, enabling their incorporation into various products, such as fruit and vegetable drinks, yogurts, and other dairy products.

Crucially, the choice of the wall material composition for each system is paramount, as the encapsulating material influences the numerous physicochemical properties of the powdered product and dictates its storage behavior [2]. An intriguing method that facilitates microencapsulation is complex coacervation. This process holds a distinctive advantage as it can occur at room temperature, a particularly noteworthy feature when dealing with essential oils [16].

Complex coacervation is one of the most important microencapsulation methods used to protect sensitive substances such as aromas, omega-3 fatty acids, vegetable oils, antioxidants, and essential oils [9,10,15,17,18]. This phenomenon takes place in an aqueous solution when two polymers with opposite charges are drawn together by electrostatic forces. Subsequently, this interaction leads to phase separation, creating two distinct phases: a concentrated coacervated phase referred to as the “continuous phase,” and a less concentrated liquid phase known as the “equilibrium solution” [11,19,20]. The coacervated phase consists of wall material deposited in a thin layer of the core material. The mixtures of proteins and polysaccharides are commonly used, and the most widely studied coacervation system is gelatin (G): gum Arabic (GA) [11,21,22]. This technique allows for high encapsulation efficiency (up to 99%) [14] and enables the encapsulation of more core material per unit mass of wall material compared with other microencapsulation techniques [12,16,17,19,20]. This process has been used to microencapsulate essential oils [9,10,14,16,19]. However, so far, it has been used to do so without first dissolving EO in the oil. Complex coacervation allows for one to obtain a high efficiency of oil encapsulation [23]. However, essential oils, as highly volatile compounds, may suffer significant losses during the spray drying or freeze drying processes [9,10,12,24].

The main aim of this research was to determine whether the classical model of complex coacervation (G:GA) could be used for the microencapsulation of essential oils. The aim of this study was to examine how varying mixing ratios of wall materials (specifically, gelatin and gum Arabic) impact encapsulation efficiency. Additionally, the research sought to explore the physicochemical properties of the resulting powders. In order to minimize the loss of essential oil (due to the increased temperature of the process), the essential oils were dissolved in the oil before being microencapsulated. The literature review shows that, so far, such a solution has not been used. The amount of research into the application of the classical model of complex coacervation to microencapsulation of essential oils is also limited.

The obtained results indicate that the use of the classical model of complex coacervation for microencapsulation of essential oils is possible. This allows a large part of EO to be retained in the microcapsule structures and gives hope for their possible use in food.

## 2. Materials and Methods

### 2.1. Materials

Gelatin (Agnex, Białystok, Poland), and gum Arabic (Warchem, Warsaw, Poland) were used as wall materials. Juniper berry (*Juniperus communis*) essential oil and black pepper peppercorn (*Piper nigrum*) essential oil (Ancient Wisdom, Sheffield, Great Britain) were firstly dissolved in soybean oil (Dary Natury, Koryciny, Poland) or grape seed oil (Basso Fedele e figli s.r.l., Avellino, Italy) and used as the core material. Essential oils were dissolved in oil at a concentration of 1% *v*/*v* to reduce the risk of their evaporation during the freeze drying process.

### 2.2. Preparation of Coacervates

As a wall material, 5% gelatin solution with 5% gum Arabic solution was used. Gelatin (G) (Agnex, Białystok, Poland) and gum Arabic (GA) (Warchem, Warsaw, Poland) were dissolved at the temperature of 50 °C. The solutions were mixed in different mixing ratios: 1:1; 1:2, and 2:1. The mass of each system was 300 g. After the solutions were mixed together, they were subjected to high shear homogenization using Ultra turrax (IKA T18 basic, Staufen, Germany) for 5 min at 15,000 rpm/min at room temperature. In the homogenization process, every variant received an addition of 10% (30 g) of either soybean oil (SBO) or grape seed oil (GSO), within which 1% of the system mass (3 g) had juniper essential oil (JEO) or black pepper essential oil (BPEO) dissolved in SBO or GSO previously. After emulsification, the pH was adjusted to 4.0 (under the isoelectric point) using 1M HCl solution (Firma Chempur, Piekary Śląskie, Poland). Each emulsion underwent a storage process at 4 °C for 24 h, followed by transfer to −20 °C for an additional 24 h. Subsequently, the samples were further transferred to −60 °C for an additional 24 h. The frozen samples were then subjected to lyophilization for a duration of 72 h at −80 °C. After this period, the lyophilized products were sieved using a laboratory sieve with a mesh size of 710 μm. The screened material was vacuum packed and stored at 4 °C for subsequent analyses.

The samples were coded for easier identification (Table 1). The mixing ratio was marked as 1—1:1, 2—1:2, 3—2:1, juniper essential oil—J, black pepper oil—B, soybean oil—S, grape seed oil—G. For example, the sample in which G/GA = 1:1 and which contains juniper essential oil dissolved in soybean oil was coded as SJ1.

### 2.3. Complex Coacervation Yield, Solid Yield, and Encapsulation Efficiency

To determine the efficiency of the complex, coacervation yield (CY) was calculated according to the equation [25]:

(1) $$\mathrm{CY}=\frac{\mathrm{CM}}{\mathrm{SM}}*100\%$$

CM—coacervate mass collected after the process,
SM—total mass of the freeze-dried powder.

To determine the losses during the freeze drying process, the solid yield (SY %) was calculated according to the equation [25]:

(2) $$\mathrm{SY}=\frac{\mathrm{PM}}{\mathrm{LCM}}*100\%$$

where:

PM—powder mass collected after freeze drying,
LCM—liquid coacervate mass, before the freeze drying process.

Encapsulation efficiency (EE) was measured according to the method described by Hernandez-Nava [26] with slight modifications. Briefly, it was calculated by measuring the surface oil (SO) and total oil (TO) of the freeze-dried powders. All measurements were completed in triplicate.

SO was determined by weighing 1 g of sample and dispersion in 30 mL of n-hexane with constant stirring (60 rpm) for 15 min. The oil phase with n-hexane was filtered into the pre-weighted round-bottom flask and evaporated on a rotary evaporator (R-100 Büchi, Switzerland). Then, the sample was stored in the oven at 105 °C for 30 min to ensure all n-hexane had evaporated. After this, the sample was left in the desiccator until it had cooled (1 h). The round-bottom flask was weighed, and the SO was calculated as:

(3) $$S_{O}={\mathrm{OM}}_{1}-{\mathrm{OM}}_{2}$$

where:

OM_1_—oil mass after extraction and evaporation of the solvent,
OM_2_—the theoretical weight of oil from the sample.

T_O_ was determined by weighing 1.5 g of the sample and spreading it in 4 mL of KCl. In the next step, 4 mL of acetone and 8 mL of chloroform were added to the sample. The sample prepared was constantly stirred (60 rpm) for 15 min and then centrifuged (5000 rpm, 5 min). The sample was separated during centrifugation. The top layer was poured, and the bottom layer containing chloroform and the oil phase was passed through a filter paper with anhydrous sodium sulfate into a weighed round-bottom flask, so the test sample remained in the falcon test tube. After that, 4 mL of double-distilled water, 4 mL of acetone, and 8 mL of chloroform were added to the residue. The sample was centrifuged again, the top layer was poured, and the bottom layer was soaked through filter paper. Then the solvent was evaporated on a rotary evaporator (R-100 Büchi, Switzerland). After that, the sample was stored in the oven at 105 °C for 30 min to evaporate the excess chloroform. After this, the sample was left in the desiccator until it was cooled down (1 h). The round-bottom flask was weighed, and the T_O_ was calculated as:

(4) $$T_{O}={\mathrm{OM}}_{1}-{\mathrm{OM}}_{2}$$

where:

OM_1_—oil mass after extraction and evaporation of the solvent,
OM_2_—the theoretical weight of oil from the sample.

Having the results for S_O_ and T_O_, EE (%) was calculated according to the formula:

(5) $$\mathrm{EE}=\frac{T_{O}-S_{O}}{T_{O}}*100\%$$

### 2.4. Bulk and Tapped Density, Carr Index (CI), and Hausner Ratio (HR)

The bulk density (ρ_bulk_) was determined by measuring the volume occupied by a known quantity of powder in a 10 mL measuring cylinder, measuring its weight before and after adding the powder [26]. Similarly, the tapped density (ρ_tap_) was determined in a 10 mL measuring cylinder by repeatedly tapping manually by lifting and dropping the cylinder under its own weight at a vertical distance of 14 ± 2 mm high for one minute (one tap per second) [27]. Determinations were made in triplicate. Bulk and tapped densities were expressed in g/cm^3^.

The compressibility index (CI) and Hausner ratio (HR) are measures of the tendency of a powder to be compressed. Determinations were made in triplicate. To calculate the CI and HR, ρ_bulk_ and ρ_tap_ were used in these equations [28]:

(6) $$\mathrm{CI}=\frac{\rho_{\mathrm{tap}}-\rho_{\mathrm{bulk}}}{\rho_{\mathrm{tap}}}*100\%$$

(7) $$\mathrm{HR}=\frac{\rho_{\mathrm{tap}}}{\rho_{\mathrm{bulk}}}$$

### 2.5. Color Measurement

The color of the microcapsules was examined using a Minolta CR-400 colorimeter (Konica Minolta Inc., Tokyo, Japan) (D65 illuminate, measuring surface—8 mm, standard 2° observers). The measurement results are expressed following the system of the International Commission on Lighting (Comission Internationale de L’Eclarige) in the CIELab color space. The following parameters were determined and tested: L* (L = 0 (black), L = 100 (white)), a* (−a = green, +a = red), and b* (−b = blue, +b = yellow) [29]. Determinations were made in triplicate immediately after production.

### 2.6. Solubility, Hygroscopicity, and Moisture Content

The solubility of samples was measured according to the method described by Shaddel et al. [30]. Briefly, 0.5 g of freeze-dried sample was weighed into a falcon test tube containing 50 mL of double-distilled H_2_O. The sample prepared in this way was homogenized for 30 min and then centrifuged (5000 rpm, 5 min). After centrifugation, 25 mL aliquot of the supernatant was transferred into a dried and pre-weighed Petri dish and immediately oven dried at 105 °C for 6 h. Then, the solubility (%) was calculated by weight difference. Determinations were made in triplicate.

The hygroscopicity of samples was measured according to the method described by Shaddel et al. [30]. Briefly, 0.2 g of each freeze-dried sample was placed in a Petri dish and stored in a container containing the saturated solution of Na_2_SO_4_ for one week. Hygroscopicity was expressed as g of water absorbed per 100 g sample (%). Determinations were made in triplicate.

The moisture content of microcapsules was determined gravimetrically by drying in a Binder FP 115 drying oven (Binder, Tuttlingen, Germany) at 70 °C for at least 24 h, then cooling to room temperature in desiccators until a constant weight was reached. Determinations were made in triplicate.

### 2.7. Particle Size Distribution

The measurement was carried out with the Morphologi^®^ G3SE apparatus (Malvern Instruments Ltd., Malvern, UK) equipped with a dispersion unit for dry samples. The particle size distribution was calculated as the relative volume of molecules in the band, shown as size distribution curves (Malvern Microsoft ware v.5.40, Malvern Instruments Ltd.). The examined parameters of the size distribution contained the largest particle size (D (v, 0.9)), mean particle volume (D (v, 0.5)), and the smallest particle size (D (v, 0.1)) [31]. The particle size distribution (Span index (SI)) was estimated using the following formula [32]:

(8) $$\mathrm{SI}=\frac{D_{90}-D_{10}}{D_{50}}$$

### 2.8. Fourier Transform Infrared Spectroscopy (FT-IR)

FT-IR spectra were recorded on a Nicolet™ iS™ 5 FTIR Spectrometer (Thermo Scientific, Waltham, MA, USA), with horizontal device for attenuated reflectance and diamond crystal, on a spectral window ranging from 4000 to 400 cm^−1^, at a spectral resolution of 2 cm^−1^. Spectra were recorded without any sample preparation and were processed with the OMNIC program (Thermo Scientific, Waltham, MA, USA).

### 2.9. Differential Scanning Calorimetry (DSC)

The thermal properties of the samples were evaluated using differential scanning calorimetry (DSC 1) from Mettler Toledo (Schwerzenbach) under an argon atmosphere at a flow rate of 100 cm^3^/min, as per the method described by Zhao et al. [33] with some modifications. The instrument was calibrated with pure indium and zinc. Each sample (5.0 ± 0.1 mg) was placed in a standard 40 μL aluminum crucible (ME-51119870) and covered with a lid (ME-51119871) using the Mettler Toledo Crucible Sealing Press. DSC scans were recorded from 10 °C to 230 °C at a rate of 10 °C/min (β). The thermograms were analyzed using STARe Software (Version 9.30) to determine the start (T_on_), maximum (T_max_), and end (T_end_) temperatures, as well as the areas under the peaks (ΔH).

### 2.10. Electronic Nose Analysis

Volatile compounds within the microcapsules were extracted using the Heracles II electronic nose (Alpha M.O.S., Toulouse, France) following the methodology outlined in the works of Wojtasik-Kalinowska et al. [34] and Górska-Horczyczak et al. [35]. This electronic nose employs ultra-fast gas chromatography with headspace and features a detection system comprising two metal columns of varying polarities (nonpolar MXT-5 and slightly polar MXT1701, diameter = 180 µm, length = 10 m) and two flame ionization detectors (FID). Kovats indexes were established using alkane standards (n-butane to n-hexadecane) (Restek) measured under the same conditions as the samples. Identification of volatile compounds was accomplished using the AroChemBase (Alpha MOS Co., Toulouse, France), containing 44,000 compounds and a base of sensory descriptors for each compound.

For analysis, 10% solutions of each sample were placed in 20 mL headspace vials sealed with a Teflon-faced silicon rubber cap. These vials were then incubated at 35 °C for 900 s under an agitation speed of 8.33 Hz. Hydrogen was used as the carrying gas at a constant flow rate of 1 mL/min. The injector temperature was set at 200 °C, with an injected volume of 3500 µL and an injection speed of 125 mL s^−1^. The analytes were collected in the trap at 15 °C and subsequently divided and simultaneously transferred into the two columns. The carrying gas was maintained at a constant pressure of 80 kPa, with a split flow rate of 10 mL min^−1^ at the column heads. The temperature program in the oven was as follows: 60 °C for 2 s, a ramp of 3 °C s^−1^ to 270 °C, held for 20 s, and FID1/FID2 at 280 °C. Flavor data profiles were presented through principal component analysis (PCA) using AlphaSoft Version 8.0 software. All samples were analyzed in triplicate.

### 2.11. Scanning Electron Microscopy Analysis (SEM)

A scanning electron microscope (Jeol JSM6010LA, Tokyo, Japan) was employed to analyze the samples’ surface morphologies after the different treatments. Each sample was fixed on an adhesive carbon tape and coated (approximately 15 nm) with gold. Secondary electron images were taken at several magnifications and an accelerating voltage of 10 kV.

### 2.12. Statistical Analysis

For statistical analysis, the STATISTICA computer program was used. To check significant differences between the results, one-way and multivariate analyses of variance ANOVA and the Fisher LSD test (*p*-value < 0.05, α = 95%) were used.

## 3. Results

### 3.1. Evaluation of Coacervation Efficacy

The critical factor influencing the complex coacervation process is the protein-polysaccharide mass ratio. Different ratios impact the intensity of interaction and complexation due to the charge balance between protein and polysaccharide [26,36,37]. The statistical analysis demonstrated that the obtained results had been affected by the mixing ratio, essential oil, the type of oil used, and their interactions. However, the mixing ratio had the most significant impact on the resulting coacervation yield (CY) and solid yield (SY), closely followed by the essential oil (EO) (Table 2). The mixing ratio exhibited a strong positive effect on CY but had a negative impact on SY. Similarly, the type of oil had a small effect, with a slight decrease in CY and a slight increase in SY values. Notably, the content of essential oil caused an opposite effect—it led to a slight decrease in CY and a slight increase in SY values. In this study, both CY and SY values were relatively low, not exceeding 50%. Samples GJ3, GB3, SJ3, and SB3 exhibited the highest CY values, with significant differences (*p* < 0.05) among them. Despite having the highest CY values, these samples did not reflect the highest SY. The highest SY values were observed for samples SB2, GB2, GJ2, and SB1, characterized by the lowest CY values. The specific molar mixing ratio for the achievement of maximum coacervate yield occurs when protein and polysaccharide have the exact opposite charge density, leading to charge neutralization [14,38,39]. This likely occurred at a mixing ratio of 2:1, where the ratio of gelatin to gum Arabic was the highest, resulting in the highest CY. Gelatin, rich in positively charged NH3+ ions, and gum Arabic, with primarily negatively charged COO- ions, undergo electrostatic attraction in the coacervation process [40]. Despite gelatin’s high water-binding capacity, these polymers form weak hydrogen bonds with water, which disintegrate and release water during the freeze drying process [41].

Encapsulation efficiency (EE) represents the percentage of the core material enclosed within the powder particles, a critical parameter for essential oils given their volatility and susceptibility to loss during the drying process. Statistical analysis revealed that the results had been significantly influenced by the interaction between the mixing ratio of polymers and oil (Table 2). The type of oil exhibited a notable positive impact on EE, leading to an increase in its values. The highest EE was observed for sample SJ1 (64.09% ± 0.09). Additionally, notable encapsulation efficiency was achieved for samples SB3 and GJ2 (61.92 ± 0.04 and 59.89 ± 0.01, respectively), with significant differences (*p* < 0.05) in EE. Samples GB2, SB1, and SJ2 did not show significant differences (*p* < 0.05) in EE, with values falling within the middle of the obtained range (53.4–55.25%). Notably, similarities were identified in samples SB1 and GB2, which shared the same oil and essential oil, as well as in samples GB2 and SJ2, featuring the same mixing ratio (1:2). The influence of the interaction between mixing ratio and oil on the EE value is evident in these cases. Samples GJ1 and GJ3 were also within the same statistical group (49.30 ± 0.07 and 49.65 ± 0.02, respectively), containing the same essential oil. The lowest EE value was recorded for sample GB1 (Table 2).

### 3.2. Bulk Density, Tapped Density, Carr Index (CI), and Hausner Ratio (HR)

Bulk density (ρbulk) is defined as the ratio of the mass to the volume (including the inter-particle void volume) of an untapped (loose) powder sample. If the bulk density is high, the volume of the powder is lower; when the bulk density is low, the same powder mass takes up a larger volume. The tapped density (ρtap) is defined as an increased bulk density attained after mechanically tapping a cylinder containing the powder sample. This property influences the appropriate selection of the size of the containers for packing the powder (e.g., barrels, bags). Analysis of bulk and tapped density of powder gives a possibility to calculate the compressibility index (Carr index) and Hausner ratio of the powder [28].

The Carr index (CI) serves as an indicator of powder bridge strength and stability, while the Hausner ratio (HR) reflects interparticulate friction [42]. These metrics are instrumental in assessing the flow characteristics of powders. Powder bridging takes place when particles interlock or bond, forming an arch above the container outlet. The cohesive strength and internal friction of individual particles together play a crucial role in determining the stability and strength of this arch. In instances where a powder experiences bridging, the arch effectively holds the remaining contents within the container, impeding the discharge of the remaining powder. A powder’s lower CI or lower HR indicates better flow properties than higher ones. A CI of <10 or HR of <1.11 is considered ‘excellent’ flow, whereas CI > 38 or HR > 1.60 is considered ‘very very poor’ flow [43]. In CI and HR, the obtained values were influenced by the mixing ratio and its interaction with the oil. The increase in the mixing ratio resulted in a decrease in the cohesiveness of the obtained powders, and thus a decrease in the CI and HR values. The samples differed statistically significantly (*p* < 0.05). The GB2, GB3, SJ2, and SB2 samples were in the same statistical group (Table 3), presenting the CI in the range of 31.81–34.74 and the HR in the range of 1.47–1.53, thus demonstrating high cohesiveness and very poor flow [44]. These were also the lowest CI and HR values obtained in this study. The highest values were recorded for the GJ1 and GB1 samples, which were also together in the same statistical group (*p* < 0.05). CI values were 50.27 ± 2.53 and 48.84 ± 7.73, respectively, and HR values were 2.01 ± 0.11 and 2.98 ± 0.28, respectively. These powders had the highest cohesiveness and virtually no flow [45].

The powders obtained in this study were characterized by a very low bulk density in the range of 0.11–0.17 and a tapped density from 0.22 to 0.28 (Table 3). The tapped density exceeded the bulk density because smaller particles filled the voids between larger particles, achieving a more compact packing condition due to the tapping process [46]. The greatest influence on the obtained bulk density was the mixing ratio and the interaction between the mixing ratio, oil, and essential oil. All factors caused an increase in bulk density. The samples showed statistically significant differences among themselves (*p* < 0.05). Similarly, in the case of tapped density, the mixing ratio and interactions between it and the oil and essential oil had the greatest impact on the obtained results. The influence of the mixing ratio was so positive that it caused the tapped density value to increase as it increased. Oil also had a slight positive effect while EO had a slight negative effect. The samples differed statistically significantly (*p* < 0.05).

### 3.3. Solubility, Hygroscopicity, and Moisture Content

In the food industry, the solubility of powders in water is a crucial factor to expand their range of applications. However, complex coacervation aims to produce water-insoluble microcapsules, allowing for controlled release of the core material. In this study, all samples exhibited low solubility in water (>26%) (Table 3). The solubility of the powders was primarily influenced by the mixing ratio of polymers, as well as the interaction between the mixing ratio and the oil, and the interaction between the mixing ratio and essential oil (*p* < 0.05). These interactions led to a decrease in the solubility of the powders. The least soluble samples were GJ3, GB3, and SB3, forming a homogeneous statistical group with solubility below 8%. The impact of the mixing ratio and the oil used to dissolve the essential oil is evident here, with sample SB2 showing the highest solubility (25.86% ± 6.81).

Hygroscopicity is a critical factor affecting the stability of food products, storage conditions, and packaging materials. Powders with hygroscopic values exceeding 15.1% are considered highly hygroscopic [47]. However, the hygroscopicity of the powders in this study ranged from 1.49% to 7.29% (Table 3), indicating low hygroscopicity. The examined factors did not have a statistically significant influence on the hygroscopicity of the obtained powders (*p* < 0.05), with no statistical differences found between samples.

Moisture content is another important property related to storage stability and shelf life. The moisture content in the obtained powders was low, ranging from 0.05% to 0.56% (Table 3). Similar to hygroscopicity, the examined factors did not have a statistically significant influence on the moisture content of the powders (*p* < 0.05), and no statistical differences were observed between samples.

### 3.4. Color Measurement

The color of the resulting powders was primarily influenced by the oil used to dissolve the essential oil, with grape seed oil imparting a slight greenish tint and soybean oil exhibiting a vivid yellow hue. However, the powder color was also affected by the mixing ratio, essential oil, and the interactions among these factors.

Statistical analysis revealed significant differences (*p* < 0.05) in each of the color components among the samples (Table 4). Regarding the brightness of the samples (L*), the variations were not substantial, with L* parameter values ranging from 78.72 ± 0.34 to 88.52 ± 0.25 for samples containing soybean oil and grape seed oil. Statistical homogeneity was determined for GJ3, GB3, and SJ1 samples, as well as for SJ2 and SB2. The a* parameter, indicative of the green color, exhibited negative values for all samples. For samples containing grape seed oil, the a* values ranged from −1.19 ± 0.02 to −3.83 ± 0.15. The mixing ratio played a significant role in influencing the a* value, with an increase corresponding to a higher proportion of green color. Samples containing soybean oil showed a* values ranging from −7.06 ± 0.27 to −7.66 ± 0.05, and a homogeneous group was identified for samples SB1 and SB2. In terms of the b* parameter, representing the yellow color, positive values were observed for all samples. For samples containing grape seed oil, b* ranged from 5.61 ± 0.04 to 12.3 ± 0.47, while for those with soybean oil, the range was 41.87 ± 1.05 to 51.71 ± 0.47.

### 3.5. Particle Size Distribution

The size of microcapsules should be in the range of 0.1 μm to 100 μm [12]. The particle sizes in all tested trials met these assumptions. The obtained results depend on all examined factors individually and their interactions. However, the mixing ratio of polymers had the greatest influence (*p* < 0.05). The highest values of the SI characterized the GJ3, GB3, SB3, and SJ3 samples. This means they had the highest diversity in terms of particle size (Table 5) [48]. In sample GJ3, the SI was the closest to 1.0 (SI = 0.96), so this powder contained particles with the most uniform size distribution in the tested ranges [48]. The smallest range of particle sizes and the smallest SI value were found for samples SB2 and GB2 (D_10_ = 59.04 ± 1.18 μm, D_90_ = 96.72 ± 0.5 μm, SI = 0.43 ± 0.01 and D_10_ = 60.13 ± 0.74 μm, D_90_ = 96.86 ± 0.41 μm, SI = 0.44 ± 0.01, respectively). The smallest powder particles were obtained for a mixing ratio of 1:1 (GB1, SJ1, SB1). However, no clear relationship was observed between the increase in the content of one polymer and the size of the obtained powder particles.

### 3.6. FT-IR

The obtained FT-IR spectra for JEO showed characteristic peaks at 2917.56 cm^−1^, 2878.29 cm^−1^, 1446.07 cm^−1^, 887.14 cm^−1^, 786.52 cm^−1^, and 418.96 cm^−1^. For BPO, characteristic peaks occurred at 2954.86 cm^−1^, 2922.95 cm^−1^, 2867.06 cm^−1^, 1446.24 cm^−1^, 885.83 cm^−1^, 875.20 cm^−1^, 786.32 cm^−1^, 543.65 cm^−1^, 421.49 cm^−1^, and 442.11 cm^−1^. Based on the obtained results, the compositional similarity of both essential oils can be seen, which was confirmed by the analysis of volatile compounds using the e-nose (3.8).

In the FT-IR spectra for wall materials, characteristic peaks were observed at wavenumber 3300.05 cm^−1^, 2893.28 cm^−1^, 1596.88 cm^−1^, 1413.05 cm^−1^, 1017.17 cm^−1^, 448.52 cm^−1^, 437.30 cm^−1^ and 416.39 cm^−1^ for gum Arabic and at 1627.16 cm^−1^, 1526.30 cm^−1^, 1444.40 cm^−1^, 1332.90 cm^−1^, 1235.97 cm^−1^, 1078.27 cm^−1^, 597.00 cm^−1^, 507.22 cm^−1^, 490.10 cm^−1^, 469.20 cm^−1^, 448.82 cm^−1^, 416.66 cm^−1^, 409.46 cm^−1^ and 402.23 cm^−1^ for gelatin. Regular soy oil contains approximately 54% linoleic acid (18:2), 23% oleic acid (18:1), 11% palmitic acid (16:0), 8% linolenic acid (18:3), and 4% steric acid (18:0) [49]. Grape seed oil contains the same fatty acids in similar amounts, hence the similarity of the obtained FT-IR spectra [50]. Characteristic peaks were observed at wavenumber 3008.09 cm^−1^, 2922.44 cm^−1^, 2852.97 cm^−1^, 1742.88 cm^−1^, 1457.23 cm^−1^, 1377.09 cm^−1^, 1159.43 cm^−1^, 1097.51 cm^−1^ and 721.59 cm^−1^ for grapeseed oil. For soybean oil characteristic peaks were found at 3008.58 cm^−1^, 2922.40 cm^−1^, 2852.84 cm^−1^, 1742.92 cm^−1^, 1456.95 cm^−1^, 1377.02 cm^−1^, 1159.01 cm^−1^, 1097.76 cm^−1^, and 720.99 cm^−1^.

For gum Arabic a broad absorption band was observed at wavenumber 3300.05 cm^−1^ which corresponds to hydrogen bond. Because this was followed by the presence of spectra at the 1600–1300 cm^−1^ (1596.88 cm^−1^, 413.05 cm^−1^) frequencies, we can confirm the existence of a hydroxyl (-OH) group. The next narrow band was found at 2893.28 cm^−1^ followed by peaks between 1470–720 cm^−1^, which can correspond with absorption band for long-chain linear aliphatic compounds. A characteristic peak at 1017.17 cm^−1^ corresponds to the fingerprint of carbohydrates, which, along with peaks at 448.52 cm^−1^ and 437.30 cm^−1^, is characteristic for CCO, COC, symmetrical, and asymmetrical ring breathing vibration. For gelatin, characteristic peaks were found at 1627.16 cm^−1^, which corresponds to COO asymmetric stretching, and at 1526.30 cm^−1^ and 1444.40 cm^−1^ which can be associated with COO symmetric stretching. Peaks at 1332.90 cm^−1^, 1235.97 cm^−1^, and 1078.27 cm^−1^ can be attributed to C=O stretching. The remaining characteristic peaks at 597.00 cm^−1^, 507.22 cm^−1^, 490.10 cm^−1^, 469.20 cm^−1^, 448.82 cm^−1^, 416.66 cm^−1^, 409.46 cm^−1^ and 402.23 cm^−1^ are CCO, COC, symmetrical and asymmetrical ring breathing vibrations [51].

**Figure 1:**
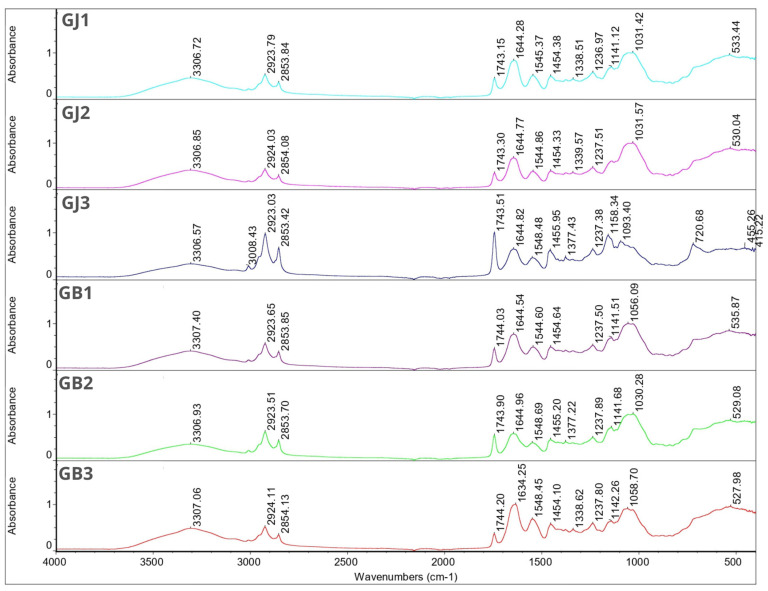
FT-IR spectra for samples GJ1, GJ2, GJ3, GB1, GB2 and GB3.

**Figure 2:**
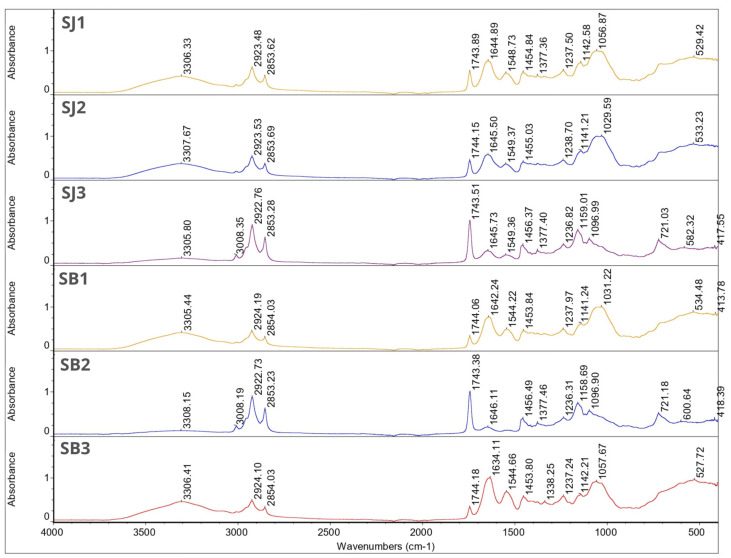
FT-IR spectra for samples SJ1, SJ2, SJ3, SB1, SB2 and SB3.

Figure 1 and Figure 2 show the FT-IR spectra for individual microcapsules. The spectra of the microcapsules were similar to those of the wall materials. Most of the peaks corresponding to the essential oils disappeared in these spectra. This phenomenon can be related to the overlapping of the peaks of the matrix, oils, and essential oils, which is due to the low concentration of essential oils in the total weight of the microcapsules [24]. Nevertheless, the obtained results confirm that both oils were successfully encapsulated. This is evident from the characteristic peaks at 1455.95 cm^−1^, 1454.10 cm^−1^, 1454.84 cm^−1^ and 1453.80 cm^−1^ seen in samples GJ3, GB3, SJ1 and SB3, respectively. It is also worth noting that samples SJ1 and SB3 have the highest encapsulation efficiency among the remaining samples.

In all samples, a characteristic broad absorption band between 3305.44–3308.15 cm^−1^ can be seen. This is characteristic for gum Arabic and hydroxyl groups [52]. For samples SB2, SJ3 and GJ3 this peak is followed by characteristic narrow peaks between wavenumber 3008.19–3008.43 cm^−1^ which can be linked with unsaturated compounds or aromatic rings characteristic for essential oils. Those peaks are not visible in other sample spectra. For all samples, peaks between 2922.73–2924.19 cm^−1^ and 2853.23–2854.13 cm^−1^ were found. Since it was found in the single bond area those peaks can contribute to long-chain aliphatic compounds [53]. There were no characteristic peaks in the triple-bond region (2000–2500 cm^−1^). In the double-bond region (1500–2000 cm^−1^) for all samples, peaks were found between 1743.15–1744.20 cm^−1^. Those peaks can describe carbonyl compounds such as aldehydes, ketones or esters characteristic for essential oils, at the same time being a proof for successful EOs encapsulation. These peaks for samples SB2, SJ3 and GJ3 were much more intense compared with the rest of the graphs, which may indicate a higher content of essential oils in these samples (according to the EE% results). In the same region we found peaks at 1634.11–1646.11 cm^−1^, which might be because of the presence of unsaturated bonds, probably C=C. The rest of the peaks are characteristic for the fingerprint region (600–1500 cm^−1^)—those peaks can contribute to the methylene C-H bond (1485–1445 cm^−1^), methyne C-H bond (1350–1330 cm^−1^), C-O stretch (~1200 cm^−1^, ~1150 cm^−1^), and CN stretch (1210–1150 cm^−1^). Again, only for samples SB2, SJ3 I GJ3 were characteristic peaks at 720.68–721.18 cm^−1^ found and these can be linked with the aromatic ring coming from essential oils [53].

### 3.7. DSC

DSC analysis was performed to verify the thermal behavior after encapsulation of the essential oils. In the curves showing the thermal behavior of the wall materials (gelatin, gum Arabic), an endothermic peak can be seen in each case—60.58 ± 0.002 °C (−1430.91 ± 0.001 mJ) for gelatin and 137.93 ± 0.001 °C (−696.52 ± 0.001 mJ) for gum Arabic. Those peaks probably refer to a glass transition. DSC curves for juniper and black pepper essential oils show an endothermic event at 24–25 ± 0.001 °C (−80.00 ± 0.001 mJ and −87.04 ± 0.002 mJ, respectively) related to the residual water and at 150 ± 0.001–158 ± 0.001 °C (−350.87 ± 0.001 mJ and −367.14 ± 0.001 mJ, respectively) related to the decomposition of JEO and BPO [24]. Table 6 shows DSC onset, peaks and endset temperatures with corresponding enthalpies for all designed microcapsules. Onset and endset temperatures for GJ1, GJ2, and GJ3 were found to be 63.67 ± 0.002–87.18 ± 0.002 °C, 56.93 ± 0.002–161.87 ± 0.002 °C and 68.75 ± 0.001–87.94 ± 0.002 °C, respectively, with different enthalpy values (−36.31 ± 0.001 mJ, −14.00 ± 0.001 mJ and −20.52 ± 0.002 mJ, respectively). Onset and endset temperatures for GB1, GB2 and GB3 were found to be 46.21 ± 0.001–113.29 ± 0.001 °C, 83.46 ± 0.001–99.21 ± 0.001 °C and 94.72 ± 0.001–151.31 ± 0.002 °C, respectively, with the following respective enthalpy values: −222.81 ± 0.001 mJ, −14.00 ± 0.001 mJ and −327.77 ± 0.001 mJ. For samples SJ1, SJ2 and SJ3 onset and endset temperatures were 55.23 ± 0.001–175.95 ± 0.001 °C, 82.32 ± 0.001–108.89 ± 0.001 °C and 68.86 ± 0.001–89.36 ± 0.001 °C, respectively. Each sample exhibited unique enthalpy values, indicative of the heat absorbed during thermal transitions. The respective enthalpy values were: −186.11 ± 0.001 mJ, −51.25 ± 0.001 mJ and −0.45 ± 0.002 mJ. Similarly, for samples SB1, SB2 and SB3, onset and endset temperatures were 59.94 ± 0.001–178.75 ± 0.001 °C, 70.13 ± 0.001–84.80 ± 0.001 °C and 71.79 ± 0.001–161.17 ± 0.001 °C, respectively, with enthalpy values of −285.82 ± 0.002 mJ, −14.50 ± 0.001 mJ and −210.46 ± 0.001 mJ, respectively. Due to the lack of peaks characteristic of JEO and BPO, it can be assumed that these essential oils have been successfully encapsulated in the wall material [24]. In terms of thermal stability, samples containing soybean oil were found to be the most stable. Additionally, the samples with the G/GA ratio of 1:1 were characterized by the highest onset temperature. From this it can be concluded that those samples would be the best variant of EO microcapsules, allowing their use in processes requiring the use of elevated temperature (not higher than 68 °C).

### 3.8. Electronic Nose Analysis

**Figure 3:**
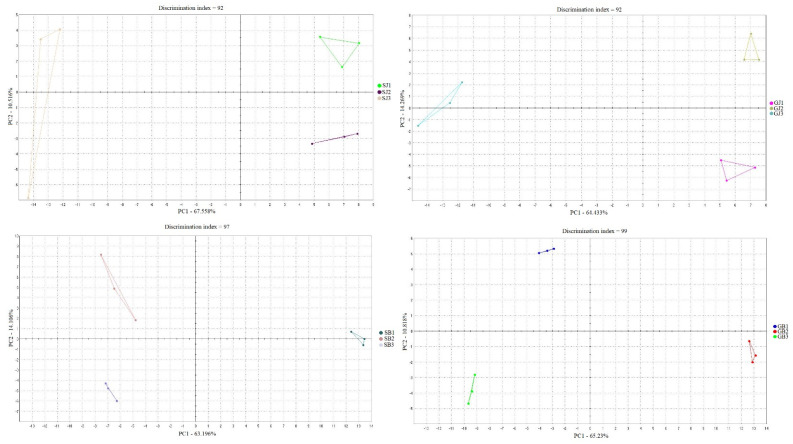
Classification of scent profiles of microcapsules containing juniper and black pepper essential oils.

Figure 3 presents the classification of scent profiles in relation to their experimental groups. Samples are represented in a two-dimensional plane with reference to selected components: principal component 1 and principal component 2. When considering essential oils separately, it can be concluded that, in the case of grape seed with juniper as an essential oil, the total contribution variances of PC1 and PC2 for direct electronic nose measurements were 60.6% and 14.27%, respectively. The combination of grape seed and black pepper as essential oil resulted in values explaining 65.23% of the data variance, where 10.81% was intercepted by the horizontal axis, explaining differences among samples along that axis. For soybean with juniper, the total contribution variances of PC1 and PC2 for direct electronic nose measurements were 63.21% and 14.10%, respectively. Soybean with black pepper caused a 67.56% data variance, with 10.51% intercepted by the horizontal axis. Additionally, there was no overlap between the mixing ratios of the single samples for all essential oils.

Table 7 lists the Kovats indexes along with the volatiles identified in each sample and their corresponding sensory descriptors. According to literature data, juniper (*Juniperus communis*) essential oil contains mainly α-pinene (25.3%), β-pinene (2.98%), sabinene (5.99%), β-myrcene (12.6%), α-terpinene (0.21%), γ-terpinene (0.80%), and limonene (2.99%) [7]. All of the abovementioned compounds except for sabinene and α-terpinene were identified in all of the samples containing juniper essential oil. These two compounds are highly volatile, and their absence in the samples can be explained by their probable evaporation during the freeze drying process [54]. Samples containing EO from juniper dissolved in soybean oil were also characterized by the content of 3-methyl-octane, 2-butylfuran and dimethyl disulfide. In turn, those with grape seed oil were the only ones that contained 3-methyl−2-butene-1-thiol, geranial and thymol. All JEO capsules contained nonane and propyl nonanoate. From this it can be seen that the oil in which the EO is dissolved has an effect on the composition of the EO volatiles after freeze drying. Similarly, in the samples containing black pepper essential oil, the compounds characteristic of this oil were identified as α-pinene, β-pinene, myrcene, and limonene. Sabinene and β-caryophyllene were not identified [55]. In addition, 5-pentanolide and isoamylacetate were identified in samples containing BPO.

In each of the samples, ethanol and 2-propanol were identified, which are probably residues from the extraction process [56].

### 3.9. SEM

**Figure 4:**
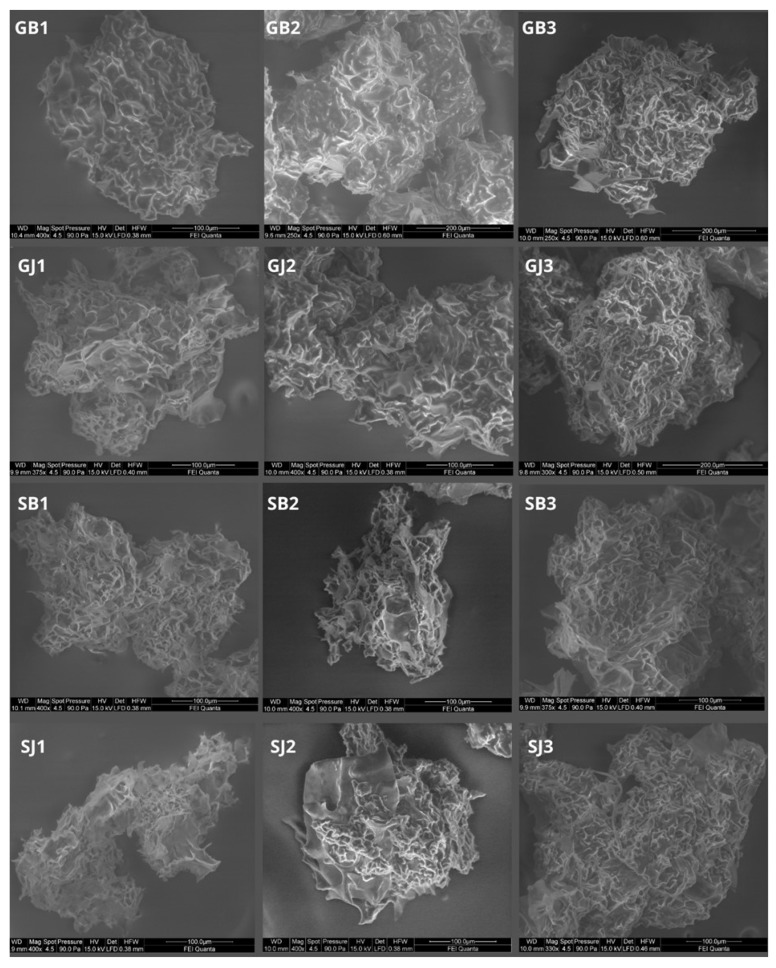
SEM photos.

Figure 4 shows SEM images of lyophilized microcapsules. As described earlier, the encapsulation efficiency was not very high (42.7–64.09%). This fact can be explained by the morphology of the microcapsules. The obtained powder was characterized by an irregular, very porous structure with a large surface area, suggesting that the core material was not completely covered. For this reason, oxygen availability can be high, causing oxidation of both the oil and the essential oil [51]. In addition, the highly porous surface of the microcapsules may make it easier for the essential oil to evaporate during storage. An increase in the concentration of polymers can make the wall of the microcapsule thicker, which can have a direct effect on the size of the resulting capsules [54]—in figures 12–15 it is clear that the microcapsules containing G:GA = 1:1 were smaller than those where the concentration of G:GA was 1:2 or 2:1.

## 4. Discussion

### 4.1. Evaluation of Coacervation Efficacy

Karagozlu [57], using a complex coacervation between gelatin and acacia, obtained a SY of about 26.66%. Hernandez-Nava [26] used G and GA as wall material (mixing ratio 2:1) and obtained an SY in the 72–88% range. These differences may be caused primarily by the difference in pH during the coacervation process and the difference in the selection of the drying method.

The most common method of encapsulating essential oils is spray drying with the use of various types of carriers—whey protein (WP), maltodextrin (MD), inulin (IN), gelatin, gum Arabic, various types of modified starches (MS), etc. The use of this method allows for encapsulation efficiencies at levels of 47.13% for WPI, 61.64% for WPI:MD, 48.14% for WPI:IN [12], 87.5–94.5% for G:GA (depending on the air temperature used at the inlet) [26] or even 98.87% for MD:MS [42]. The encapsulation efficiency for spray drying depends largely on the wall materials used, as is the case with complex coacervation. Here, however, the mixing ratio of the polymers is also important. It is well known that encapsulation efficiency increases with increasing polymer concentration, however, this is not the only factor affecting EE. The interactions between the polymers used and the amount of core material also play an important role, as shown by the results of our research. Manaf et al. [44] used simple and complex coacervation to encapsulate citronella EO. For simple coacervation they used GA (12,5% *w*/*v*), for complex coacervation they used G:GA (mixing ratio 1:1, 12,5% *v*/*w*), and in both cases they used 1% of core material. The encapsulation efficiencies of the system prepared in this way were 93.91% and 94.42%, respectively. The team did not use any method of drying the obtained coacervates, liquid forms were used for the research. Various concentrations of wall and core material were tested by Samakradhamrongthai et al. [45]. A complex coacervation with spray drying was performed between G and GA at pH = 4.0 with a mixing ratio of 1:1; 1:2, and 2:1. The results show that the lowest EE was obtained with a mixing ratio of 1:1 and a core material content of 10% (38.56%). The highest was obtained with a mixing ratio of 2:1 and a content of core material of 5% (95.15%). Much less research is available where lyophilization has been used to dry the coacervates. This method gives good results. Freeze drying of complex coacervates made between WPI and carbomethylcellulose (CMC), WPI and sodium alginate (SA) as well as WPI and chitosan (CH), has resulted in EE at the levels of 83.94%, 79.28% and 82.88%, respectively [19]. A complex coacervation with freeze drying has also been performed between G and GA [57]. The encapsulation efficiency reported was 64.31%. In our research, the encapsulation efficiency was similar to that obtained by Karagozlu et al. [57] and slightly lower compared with results obtained by Rojas-Moreno et al. [19] (max. 64.09%, min. 42.7%). To increase the efficiency of encapsulation, in our case, the amount of core material should probably be reduced (less than 10%) or the concentration of wall materials should be increased (more than 5%).

### 4.2. Bulk density, Tapped Density, Carr Index (CI), and Hausner Ratio (HR)

Bulk and tapped density depend not only on composition of the wall material, but also on the drying method. The use of WPI:IN (3:1, 1:1, 1:3) as a wall material for rosemary EO encapsulation by spray drying (170 °C) resulted in powders with bulk densities of 0.20 g/cm^3^, 0.24 g/cm^3^, and 0.26 g/cm^3^, respectively. Tapped densities were slightly higher—0.30 g/cm^3^, 0.34 g/cm^3^, and 0.41 g/cm^3^, respectively [12]. On the other hand, Yekdane et al. [58], using spray drying (170 °C), microencapsulated pomegranate seed oil and reported that the bulk density, depending on the content of pomegranate juice in the wall material, ranged from 0.4 to 0.5 g/cm^3^. Hernandez-Nava et al. [26], used the complex coacervation with spray drying between G:GA and gelatin and chia mucilage (CM) to microencapsulate oregano EO. Depending on the spray drying parameters (160 °C or 180 °C), the bulk density in their study was in the range of G:GA 0.157–0.202 g/cm^3^ and 0.234–0.282 g/cm^3^ for G:CM. In contrast, the tapped density for G:GA was 0.314–0.404 g/cm^3^, and for G:CM 0.335–0.403 g/cm^3^. However, a low bulk density may indicate a high air content in the microcapsules, which in turn may favor the negative effect of lipid oxidation. The authors of [57], investigating the bulk density of freeze-dried coacervates (G:GA) containing oregano essential oil, indicated that the bulk density of the powders thus obtained was 0.527 g/cm^3^. In our case, the bulk density was lower, probably due to different solid content.

The use of MD:WPI as wall materials for spray drying and thus encapsulation of olive oil allowed us to obtain a CI in the range of 25.61–54.70 [59]. Such a large dispersion of values was caused by different mixing ratios of polymers. Hernandez-Nava et al. [26], have reported that the powders obtained by spray drying G:GA coacervates were characterized by a Carr index of approximately 50, while the HR was approximately 2. Similarly, for the G:CM system, CI was approximately 30 and HR approximately 1.43. Another team used complex coacervation between soy protein and GA to microencapsulate chia seed oil. The obtained coacervates were spray dried (130 °C) and were characterized by CI = 35 and HR = 1.25 [23]. In turn, freeze drying of MD-based emulsions (10, 15 and 20% *v*/*w*) allowed for the obtaining of powders characterized by a CI in the range of 11.62 to 17.64 and HR in the range of 1.13–1.21 [60]. These values were much lower than in our case, where we freeze dried coacervates (CI min. 30.58, max. 50.27, HR min. 1.45, max. 2.01). Therefore, it can be concluded that the powders obtained as a result of complex coacervation are characterized by very high cohesiveness and virtually no flow.

### 4.3. Solubility, Hygroscopicity, and Moisture Content

Fernandez et al. [12] investigated the solubility of spray-dried powders containing ginger EO. Depending on the wall material used (WPI, WPI:MD, WPI:IN), the solubility of the powders was in the range of 76.94–81.58%. Another team [19], using MD:MS as a wall material for microencapsulation of orange EO (by spray drying), obtained powders with a solubility of 57.10%. Caparino et al. [27] investigated the water solubility of mango powders obtained from the emulsion with MD by spray and freeze drying. Powders obtained by spray drying had a solubility of 95.31%, while those obtained by freeze drying had a lower solubility—89.70%. Thus, the influence of the choice of method on the solubility of the obtained powder is visible—lyophilization makes the powders less soluble. In our case, the solubility was low and did not exceed 26%. These results correlate with those obtained by Shaddel et al. [30]. The powders obtained by freeze drying coacervates (5% *v*/*v* G/GA) were characterized by low water solubility, not exceeding 30%. Similarly, in the case of freeze-dried coacervates obtained by mixing G:GA in the ratio 1:1 with the addition of shrimp lipid extract [61]—the solubility of the obtained powder was low—approximately 9.6%. The above indicates that not only the method of drying, but also the type of dried material (emulsion, coacervates), has a significant impact on the solubility of powders. Powders obtained by the freeze drying of complex coacervates show much lower solubility in water than those obtained from the classical emulsion.

Zotarelli et al. [47] found that mango powders obtained by the spray drying of mango pulp with maltodextrin (MD) displayed high hygroscopicity in the range of 23.9–26.9%. In a study by Caparino et al. [27], the hygroscopicity of mango powders obtained by spray and freeze drying of mango puree with MD was reported to be around 16.5% for spray-dried samples and approximately 18% for freeze-dried samples. Similarly, in the case of the spray drying (at 170 °C) of an emulsion containing WPI:IN and rosemary essential oil, the hygroscopicity of the resulting powders varied from 15.7% to 17.1%, depending on the mixing ratio of the wall materials [32].

In contrast, powders obtained by the freeze drying of coacervates showed significantly lower hygroscopicity. Tavares et al. [62] have reported that coacervates with ginger essential oil exhibited lower hygroscopicity than coacervates without, ranging from 6.64% to 9.65%, which is below the threshold of 15.1%. This aligns more closely with the values obtained in our study. Gomez-Estaca [59] demonstrated that coacervates (G:GA, freeze dried) containing shrimp lipid extract had hygroscopicity at the level of 2.33 ± 0.12 g/100 g. Consequently, it can be inferred that both the addition of essential oil and the utilization of complex coacervation as a microencapsulation method have a positive effect on reducing the hygroscopicity of the obtained powders. Based on the results obtained and their comparison with literature data, it can be concluded that the obtained microcapsules could be stored for a longer time without compromising their properties.

The moisture content of powders obtained through various encapsulation methods and wall materials can vary significantly. Fernandes et al. [12] demonstrated that ginger essential oil (EO) powders obtained by spray drying had moisture content ranging from 1.05% to 1.98%, depending on the wall material used (WPI, WPI:MD, WPI:IN). Similar results were reported by Zotarelli et al. [47], where the moisture content of spray-dried mango powders with maltodextrin (MD) was 1.5%. Slightly higher hygroscopicity was observed in spray-dried coacervates, with moisture content ranging from 3.86% to 4.55% (G:GA) and 3.49% to 4.58% (G:CM) [26]. Rojas-Moreno et al. [19] found that the moisture content of spray-dried microcapsules containing orange essential oil varied from 1% to 4.5%, while freeze-dried samples exhibited slightly higher moisture content (2% to 5.5%). This difference in moisture content between spray-dried and freeze-dried samples could be attributed to the presence of oil droplets, which act as a vapor transport barrier, reducing the evaporation of water and increasing the hydrophobicity of the microcapsules. The freeze drying of coacervates containing ginger essential oil, with different wall materials (GA:CH, WPI:GA), resulted in powders with moisture contents of approximately 3.04% and 3.10%, respectively. The addition of essential oil significantly reduced these values, as coacervates without essential oil had moisture contents of approximately 6.01% and 8.02%, respectively [63]. The freeze drying of coacervates (G:GA) containing oregano essential oil produced powders with a water content of 3.39% [57].

The selection and concentration of wall materials play a crucial role in determining the moisture content of the obtained powders. The results obtained in our study (moisture content = 0.05% to 0.56%) are consistent with those obtained by Samakradhamrongthai et al. [45], who, by mixing gelatin and gum Arabic in a 1:1 ratio, obtained powders with moisture content ranging from 0.32% to 0.72% for different concentrations of core material. Based on the above, it can be concluded that the use of gelatin with gum Arabic in the coacervation process allows one to obtain powders with low moisture content, and that are thus suitable for long-term storage.

### 4.4. Color Measurement

Syed et al. [63] conducted color measurements of, inter alia, unrefined soybean oil. The results obtained in the study correlate with those obtained in this study in terms of the b* parameter—the researchers indicate the value of b* as 40.0 ± 0.60. There is some discrepancy in the case of the a* parameter. The team indicated positive values (4.0 ± 0.65), while the results we obtained clearly indicate negative a* values. Such a change in the a* parameter should be attributed to the wall materials used for encapsulation and to the presence of EO. Bruhl and Unbehend [64] carried out measurements of the color of grape seed oil. The L* parameter obtained by the researchers was similar to the results obtained in this study L* = 99.44. In turn, parameters a* and b* had higher values, although with the same sign: a* = −4.68, b* = 12.49. Again, the discrepancy in the results was largely caused by wall materials and essential oil.

### 4.5. Particle Size Distribution

Several studies have investigated particle size and similar parameters for powders produced by various methods, such as spray drying and complex coacervation. Rojas-Moreno et al. [19] examined powders obtained by spray drying coacervates (WPI:CMC, WPI:SA, WPI:CH), reporting particle sizes of 6.31 μm, 10.59 μm, and 9.44 μm, respectively, along with surface index (SI) values of 0.95 μm, 1.03 μm, and 10.6 μm, respectively.

Hernandez-Nava et al. [26] conducted similar studies involving complex coacervation between gelatin (G) and gum Arabic (GA). Depending on the parameters, they observed particle sizes (D10, D50, and D90) ranging from 6.08 μm to 30.31 μm. It is worth noting that spray drying generally allows for smaller particle sizes [25]. For instance, the particle size of spray-dried smoke powder food flavoring has been reported to be 6.3–6.9 μm, significantly smaller than freeze-dried preparations (134.7–580.4 μm) [65]. Freeze-dried G:GA coacervates had a particle size of 41.26 µm. Tavares et al. [63] conducted complex coacervation between WPI:CH and GA:CH, followed by freeze drying. Their results include D10 of approximately 25.06 μm, D50 of approximately 103.39 μm, D90 of approximately 293.51 μm, and an SI of approximately 2.60 for WPI:CH. For GA:CH, the results were D10 ≈ 19.18 μm, D50 ≈ 74.23 μm, D90 ≈ 189.21 μm, and SI ≈ 2.29. In our study, the screening of the obtained lyophilizates on a laboratory sieve played a crucial role in achieving size uniformity and obtaining relatively small particles, consistent with the standard for lyophilizates. This method allowed for the control and standardization of particle sizes.

Particle size stands as a crucial quality parameter in the determination of microcapsules’ application areas. It significantly influences delivery properties and the flowability of the powder, playing a pivotal role in the selection process. Complex coacervation between CH and GA [66] in the ratio 1:1, 1:2 and 2:1 has shown that the ratio of the wall material has a significant effect on the particle size of the microcapsules. The particle size for the samples was 16.39 μm, 28.98 μm, and 49.53 µm, respectively. Particle size is significantly affected by the proportion of polymer used in the wall material, and as the particle size decreases, particle–particle interactions become easier due to the increase in surface area [66].

### 4.6. SEM

Marfil et al. [67], using the same mixing ratios G:GA (1:1, 1:2, 2:1), obtained microcapsules with a completely different surface—smooth, without pores—and individual microcapsules were connected with each other via solid bridges, characteristic of freeze-dried products. This type of surface suggests that the entire core material was covered with wall material and the encapsulation efficiency should be high. Meanwhile, in the case of capsules that had the same concentration of wall materials and core material as in the present study, the encapsulation efficiency was quite similar, ranging from 50.08 to 83.5%. Differences in the obtained structure may be due to the difference in lyophilization temperature, which in the case of studies conducted by Marfil et al. [68] was higher (−40 °C) than in this study (−80 °C). As described by Krokida et al. [69]—where the porosity of the obtained product depends on the lyophilization conditions, including the temperature—the lower the temperature, the higher the porosity. The same was later confirmed by Barresi et al. [70]. Perhaps increasing the lyophilization temperature would allow for higher encapsulation efficiency in the case of this study.

## 5. Conclusions

Complex coacervation between gelatin and gum Arabic can be used to microencapsulate essential oils. However, not all of the combinations of mixing ratios were equally effective. The highest encapsulation efficiency characterized the SJ1 sample.

A relatively low EE was obtained, influenced by the interaction between the mixing ratio and the oil. A low efficiency was probably caused by a too-high concentration of core material in relation to the wall material. The highest EE value was obtained for sample SJ1 (64.09% ± 0.09). Similarly, high encapsulation efficiency was obtained for samples SB3 and GJ2 (61.92 ± 0.04 and 59.89 ± 0.01, respectively). Thus, no clear influence of the mixing ratio between gelatin and gum Arabic was demonstrated on the obtained efficiency of the encapsulation process. The powders obtained because of lyophilization of the coacervates with a 1:1 mixing ratio were characterized by the smallest particle sizes. However, this did not reflect an increase in bulk and tapped density, and did not reflect an increase in their hygroscopicity. However, the powders containing G/GA = 1:1 were the most soluble among the powders tested. The least dissolving microcapsules were obtained with a mixing ratio of G/GA = 2:1.

The aim of this research was to determine whether the classical model of complex coacervation (G: GA) could be used for microencapsulation of essential oils. These findings suggest that such a combination of wall materials can be used for this purpose. Further research should be carried out to find the optimal mixing ratio between gelatin and gum Arabic that would allow for higher encapsulation efficiency. Future investigations may also focus on optimizing all process parameters for the microencapsulation of essential oils to obtain powders with better properties.

## Figures and Tables

**Table 1:** Coding of samples.

Oil	Essential Oil	Mixing Ratio G/GA	Code
Grape seed	Juniper	1:1	GJ1
		1:2	GJ2
		2:1	GJ3
	Black pepper	1:1	GB1
		1:2	GB2
		2:1	GB3
Soybean	Juniper	1:1	SJ1
		1:2	SJ2
		2:1	SJ3
	Black pepper	1:1	SB1
		1:2	SB2
		2:1	SB3

**Table 2:** Complex coacervation yield (CY), solid yield (SY), and encapsulation efficiency (EE) values [%].

	Sample	CY%	SY%	EE%
	GJ1	30.93 ± 0.44 ^b^	29.79 ± 1.21 ^c^	49.3 ± 0.07 ^abe^
	GJ2	28.01 ± 0.16 ^a^	37.11 ± 0.49 ^e^	59.89 ± 0.01 ^cdf^
	GJ3	39.22 ± 0.16 ^g^	21.32 ± 0.25 ^a^	49.65 ± 0.02 ^abe^
	GB1	30.49 ± 0.56 ^b^	29.25 ± 1.29 ^c^	42.7 ± 0.11 ^e^
	GB2	29.45 ± 0.08 ^d^	36.23 ± 0.08 ^de^	55.25 ± 0.01 ^abcd^
	GB3	41.64 ± 0.15 ^i^	23.01 ± 0.36 ^b^	47.21 ± 0.04 ^ae^
	SJ1	33.78 ± 0.46 ^f^	26.58 ± 0.36 ^g^	64.09 ± 0.09 ^f^
	SJ2	31.91 ± 0.12 ^e^	33.8 ± 0.02 ^h^	53.4 ± 0.04 ^abcd^
	SJ3	46.58 ± 0.19 ^j^	22.02 ± 0.18 ^ab^	56.8 ± 0.01 ^bcdf^
	SB1	23.95 ± 0.16 ^c^	35.44 ± 0.47 ^d^	54.14 ± 0.05 ^abcd^
	SB2	28.34 ± 0.12 ^a^	38.45 ± 0.34 ^i^	52.33 ± 0.01 ^abc^
	SB3	41.04 ± 0.51 ^h^	20.21 ± 0.61 ^f^	61.92 ± 0.04 ^df^
	S.E.M	0.369	0.096	26.524
Effect	Oil	**	NS	**
	Essential oil	**	**	NS
	Mixing ratio	**	**	NS
	Oil × essential oil	**	**	NS
	Essential oil × mixing ratio	**	**	**
	Oil × mixing ratio	**	**	NS
	Oil × essential oil × mixing ratio	**	**	NS

Results in this table are expressed as mean ± standard deviation. Mean values with the same superscript letters within a row are not significantly different at *p* ≤ 0.05. S.E.M.—standard error of the mean. **=p≤0.001;NS—non-significant effect=p>0.05.

**Table 3:** Bulk density, tapped density_,_ Carr index, Hausner ratio [g/cm^3^], and solubility, hygroscopicity and moisture content (g/100 g).

	Sample	Bulk Density g/cm^3^	Tapped Density g/cm^3^	Carr Index g/cm^3^	Hausner Ratio g/cm^3^	Solubility g/100 g	Hygroscopicity g/100 g	Moisture Content g/100 g
	GJ1	0.12 ± 0.01 ^c^	0.24 ± 0.00 ^abc^	50.27 ± 2.53 ^d^	2.01 ± 0.11 ^d^	19.4 ± 2.92 ^be^	2.53 ± 0.32 ^a^	0.27 ± 0.04 ^a^
	GJ2	0.16 ± 0.01 ^a^	0.23 ± 0.01 ^ab^	30.58 ± 4.93 ^a^	1.45 ± 0.10 ^a^	12.54 ± 3.76 ^acd^	7.29 ± 8.02 ^a^	0.18 ± 0.12 ^a^
	GJ3	0.16 ± 0.01 ^ab^	0.24 ± 0.01 ^abc^	31.81 ± 4.45 ^a^	1.47 ± 0.09 ^ab^	7.85 ± 3.54 ^a^	2.87 ± 2.15 ^a^	0.56 ± 0.62 ^a^
	GB1	0.11 ± 0.01 ^c^	0.22 ± 0.02 ^ab^	48.84 ± 7.73 ^d^	1.98 ± 0.28 ^d^	18.22 ± 3.35 ^bd^	3.77 ± 2.51 ^a^	0.29 ± 0.42 ^a^
	GB2	0.17 ± 0.01 ^b^	0.26 ± 0.00 ^de^	33.56 ± 3.46 ^ab^	1.51 ± 0.08 ^ab^	20.7 ± 4.15 ^be^	6.98 ± 3.85 ^a^	0.05 ± 0.05 ^a^
	GB3	0.16 ± 0.01 ^a^	0.24 ± 0.01 ^abc^	34.64 ± 2.13 ^ab^	1.53 ± 0.05 ^ab^	6.58 ± 2.69 ^a^	6.00 ± 4.84 ^a^	0.36 ± 0.29 ^a^
	SJ1	0.12 ± 0.00 ^c^	0.22 ± 0.03 ^a^	44.35 ± 6.21 ^cd^	1.81 ± 0.21 ^cd^	16.39 ± 4.46 ^bcd^	4.92 ± 4.18 ^a^	0.30 ± 0.21 ^a^
	SJ2	0.16 ± 0.00 ^ab^	0.25 ± 0.00 ^cd^	34.74 ± 0.64 ^ab^	1.53 ± 0.02 ^ab^	18.72 ± 5.65 ^bd^	4.32 ± 3.06 ^a^	0.44 ± 0.66 ^a^
	SJ3	0.17 ± 0.01 ^ab^	0.28 ± 0.00 ^e^	40.28 ± 4.6 ^bc^	1.68 ± 0.13 ^bc^	11.14 ± 3.01 ^ac^	2.71 ± 1.97 ^a^	0.36 ± 0.30 ^a^
	SB1	0.14 ± 0.02 ^d^	0.23 ± 0.01 ^ab^	38.05 ± 8.82 ^abc^	1.64 ± 0.22 ^abc^	10.74 ± 2.41 ^ac^	2.63 ± 0.17 ^a^	0.24 ± 0.25 ^a^
	SB2	0.16 ± 0.00 ^ab^	0.25 ± 0.01 ^bcd^	34.33 ± 2.05 ^ab^	1.52 ± 0.05 ^ab^	25.86 ± 6.81 ^e^	2.86 ± 0.82 ^a^	0.28 ± 0.24 ^a^
	SB3	0.15 ± 0.01 ^ad^	0.24 ± 0.01 ^abcd^	37.75 ± 2.98 ^abc^	1.61 ± 0.08 ^abc^	7.47 ± 1.34 ^a^	1.48 ± 0.48 ^a^	0.49 ± 0.08 ^a^
	S.E.M	0.000	0.000	23.152	0.020	15.466	12.105	0.113
Effect	Oil	NS	NS	NS	NS	NS	NS	NS
	Essential oil	NS	NS	NS	NS	NS	NS	NS
	Mixing ratio	*	**	**	**	**	NS	NS
	Oil × Essential oil	NS	*	NS	NS	NS	NS	NS
	Essential oil × mixing ratio	*	*	*	*	*	NS	NS
	Oil × mixing ratio	NS	*	NS	NS	*	NS	NS
	Oil × essential oil × mixing ratio	*	*	NS	NS	NS	NS	NS

Results in this table are expressed as mean ± standard deviation. Mean values with the same superscript letters within a row are not significantly different at *p* ≤ 0.05. S.E.M.—standard error of the mean. *=p≤0.05; **=p≤0.001;NS—non-significant effect=p>0.05.

**Table 4:** Color parameters of obtained powders.

	Sample	L*	a*	b*
	GJ1	86.99 ± 0.32 ^cd^	−1.74 ± 0.14 ^h^	6.3 ± 0.19 ^bc^
	GJ2	88.52 ± 0.25 ^g^	−2.09 ± 0.02 ^g^	6.56 ± 0.13 ^c^
	GJ3	84.46 ± 0.97 ^a^	−3.05 ± 0.08 ^e^	9.47 ± 0.37 ^d^
	GB1	87.08 ± 0.23 ^c^	−1.19 ± 0.02 ^i^	5.61 ± 0.04 ^b^
	GB2	87.00 ± 0.11 ^c^	−2.91 ± 0.01 ^e^	9.24 ± 0.02 ^d^
	GB3	84.10 ± 0.87 ^a^	−3.83 ± 0.15 ^f^	12.30 ± 0.47 ^f^
	SJ1	84.52 ± 0.05 ^a^	−7.66 ± 0.05 ^c^	46.52 ± 0.13 ^ae^
	SJ2	85.64 ± 0.33 ^b^	−7.38 ± 0.14 ^ab^	45.82 ± 0.31 ^e^
	SJ3	78.72 ± 0.34 ^d^	−7.06 ± 0.27 ^d^	51.71 ± 0.47 ^h^
	SB1	86.07 ± 1.03 ^bc^	−7.37 ± 0.21 ^ab^	41.87 ± 1.05 ^g^
	SB2	85.70 ± 0.30 ^b^	−7.49 ± 0.11 ^ac^	46.86 ± 0.38 ^a^
	SB3	82.29 ± 0.56 ^f^	−7.2 ± 0.07 ^bd^	47.06 ± 0.64 ^a^
	S.E.M	0.302	0.017	0.201
Effect	Oil	**	**	**
	Essential oil	*	**	**
	Mixing ratio	**	**	**
	Oil × essential oil	**	**	**
	Essential oil × mixing ratio	**	**	NS
	Oil × mixing ratio	**	**	**
	Oil × essential oil × mixing ratio	*	**	**

Results in this table are expressed as mean  ±  standard deviation. Mean values with the same superscript letters within a row are not significantly different at *p*  ≤  0.05. S.E.M.—standard error of the mean. *=p≤0.05; **=p≤0.001; NS—non-significant effect=p>0.05.

**Table 5:** Particle size and particle size distribution index—span (SI) [μm].

	Sample	Particle Size μm	D_10_ μm	D_50_ μm	D_90_ μm	SI μm
	GJ1	20.48 ± 0.16 ^h^	56.79 ± 0.62 ^b^	83.42 ± 0.36 ^b^	96.84 ± 0.35 ^a^	0.48 ± 0.01 ^b^
	GJ2	9.18 ± 0.14 ^f^	53.66 ± 0.54 ^h^	81.98 ± 0.64 ^a^	95.55 ± 0.41 ^b^	0.47 ± 0.01 ^b^
	GJ3	4.51 ± 0.18 ^d^	51.9 ± 0.4 ^g^	81.7 ± 0.98 ^a^	95.37 ± 0.36 ^b^	0.96 ± 0.01 ^g^
	GB1	5.55 ± 0.29 ^a^	45.86 ± 0.33 ^f^	82.79 ± 0.75 ^ab^	95.7 ± 0.34 ^b^	0.6 ± 0.00 ^e^
	GB2	10.5 ± 0.25 ^c^	60.13 ± 0.74 ^c^	85.82 ± 1.46 ^d^	96.86 ± 0.41 ^a^	0.44 ± 0.01 ^a^
	GB3	12.74 ± 0.34 ^g^	43.14 ± 1.01 ^e^	77.77 ± 0.18 ^i^	95.38 ± 0.26 ^b^	1.17 ± 0.01 ^h^
	SJ1	5.57 ± 0.35 ^a^	22.2 ± 0.25 ^d^	72.98 ± 0.79 ^h^	92 ± 0.18 ^d^	0.53 ± 0.01 ^d^
	SJ2	6.67 ± 0.29 ^b^	57.59 ± 0.88 ^b^	84.11 ± 0.29 ^bc^	96.78 ± 0.55 ^a^	0.51 ± 0.01 ^c^
	SJ3	6.48 ± 0.46 ^b^	17.42 ± 0.5 ^a^	51.6 ± 1.16 ^e^	90.01 ± 0.14 ^b^	1.41 ± 0.02 ^j^
	SB1	5.71 ± 0.38 ^a^	17.37 ± 0.46 ^a^	68.25 ± 0.58 ^g^	97.24 ± 0.1 ^a^	0.67 ± 0.01 ^f^
	SB2	10.61 ± 0.35 ^c^	59.04 ± 1.18 ^c^	84.82 ± 0.22 ^cd^	96.72 ± 0.5 ^a^	0.43 ± 0.01 ^a^
	SB3	7.59 ± 0.34 ^e^	16.69 ± 0.25 ^a^	60.77 ± 0.94 ^f^	92.67 ± 0.14 ^e^	1.25 ± 0.02 ^i^
	S.E.M	0.094	0.437	0.630	0.118	0.000
Effect	Oil	**	**	**	**	**
	Essential oil	NS	**	*	**	**
	Mixing ratio	**	**	**	**	**
	Oil × essential oil	**	**	**	**	**
	Essential oil × mixing ratio	**	**	**	**	**
	Oil × mixing ratio	**	**	**	**	**
	Oil × essential oil × mixing ratio	**	**	**	**	**

Results in this table are expressed as mean  ±  standard deviation. Mean values with the same superscript letters within a row are not significantly different at *p*  ≤  0.05. S.E.M.—standard error of the mean. *=p≤0.05; **=p≤0.001; NS—non-significant effect=p>0.05.

**Table 6:** Temperatures of the onsets, peaks and endsets of endothermic reactions with enthalpy.

Sample	Onset (°C)	Peak (°C)	Endset (°C)	Enthalpy (mJ)
GJ1	63.67 ± 0.002	76.61 ± 0.001	87.18 ± 0.002	−36.31 ± 0.001
GJ2	56.93 ± 0.002	98.08 ± 0.001	161.87 ± 0.002	−353.88 ± 0.001
GJ3	68.75 ± 0.001	79.31 ± 0.002	87.94 ± 0.002	−20.52 ± 0.002
GB1	46.21 ± 0.001	98.60 ± 0.001	113.29 ± 0.001	−222.81 ± 0.001
GB2	83.46 ± 0.001	92.76 ± 0.002	99.21 ± 0.001	−14.00 ± 0.001
GB3	94.72 ± 0.001	121.90 ± 0.001	151.31 ± 0.002	−327.77 ± 0.001
SJ1	55.23 ± 0.001	107.75 ± 0.001	175.95 ± 0.001	−186.11 ± 0.001
SJ2	82.32 ± 0.001	89.61 ± 0.001	108.89 ± 0.001	−51.25 ± 0.001
SJ3	68.86 ± 0.001	80.62 ± 0.001	89.36 ± 0.001	−0.45 ± 0.002
SB1	59.94 ± 0.001	109.09 ± 0.002	178.75 ± 0.001	−285.82 ± 0.002
SB2	70.13 ± 0.001	78.48 ± 0.002	84.80 ± 0.001	−14.50 ± 0.001
SB3	71.79 ± 0.001	121.62 ± 0.002	161.17 ± 0.001	−210.46 ± 0.001

**Table 7:** Kovats indexes, identified volatile compounds and sensory descriptors assigned to them.

Kovats Index	Identified Volatile Compound	Sensory Descriptors	GJ1	GJ2	GJ3	SJ1	SJ2	SJ3	GB1	GB2	GB3	SB1	SB2	SB3
437	ethanol 2-propanol ethanethiol	alcoholic	+	+	+	+	+	+	+	+	+	+	+	+
508		alcoholic	+	+	+	+	+	+	+	+	+	+	+	+
519		earthy, fruity, garlic											+	
541	2-methylpropanal butanal butan-2-one	aldehydic	+	+	+	+	+	+	+	+	+	+	+	+
567		chocolate, green, malty	+	+	+	+	+	+	+	+	+	+	+	+
594		acetone, butter	+	+	+	+	+	+	+	+	+	+	+	+
608	2-methylfuran methyl propanoate acetic acid	acetone, burnt			+			+					+	
610		apple, etheral, fresh									+			
617		acetic, acidic			+								+	
618	1-propanethiol 1-butanamine 1-propanethiol	alliaceous, cabbage, onion, sween			+									
638		ammoniacal, fish										+	+	+
643		allioceous, cabbage, onion, sweet									+			
437	ethanol	alcoholic	+	+	+	+	+	+	+	+	+	+	+	+
508	2-propanol	alcoholic	+	+	+	+	+	+	+	+	+	+	+	+
519	ethanethiol	earthy, fruity, garlic											+	
541	2-methylpropanal	aldehydic	+	+	+	+	+	+	+	+	+	+	+	+
567	butanal	chocolate, green, malty	+	+	+	+	+	+	+	+	+	+	+	+
594	butan-2-one	acetone, butter	+	+	+	+	+	+	+	+	+	+	+	+
608	2-methylfuran	acetone, burnt			+			+					+	
610	methyl propanoate	apple, etheral, fresh									+			
617	acetic acid	acetic, acidic			+								+	
618	1-propanethiol	alliaceous, cabbage, onion, sween			+									
638	1-butanamine	ammoniacal, fish										+	+	+
643	1-propanethiol	allioceous, cabbage, onion, sweet									+			
658	n-butanol	alcoholic, cheese, fermented									+		+	+
667	trichloroethane	chloroform, etheral	+	+	+	+		+	+	+	+	+	+	+
673	1-methoxy-2-propanol	mild				+	+	+						
684	pentan-2-one	acetone, banana, etheral				+	+	+				+	+	+
700	pentan-2-ol	alcoholic, etheral, fermented	+	+	+							+	+	+
700	heptane	alkane, fruity									+			
735	dimethyl disulfide	cabbage, cheese, garlic				+	+	+						
735	butanethiol	coffee, garlic	+	+	+									
742	(e)-2-pentanal	apple, fruity, green									+			
745	propanoic acid	cheese, fruity	+	+	+	+	+	+	+			+	+	+
759	pentanol	alcoholic, anise, balsamic												+
767	3-methylbut-2-en-1-ol	herbaceous, lavender					+	+						
780	toluene	caramelized, etheral, fruity	+	+	+				+	+	+	+	+	+
767	2-methylpentane	-				+	+	+						
792	hexanal	acorn, aldehydic, fatty				+	+	+	+	+	+	+	+	
801	ethyl butyrate	acetone, banana	+	+	+	+		+						
812	octane	alkane, fruity, fusel			+	+	+	+				+	+	
810	(e)-2-octene	-								+	+			
847	4-ethylheptane	-				+	+	+						
847	3-methyl-2-butene-1-thiol	amine, leek, onion	+	+	+									
852	methyl pentanoate	apple, etheral, fruity, green									+			
866	ethyl isovalerate	anise, apple, blackcurrant	+	+	+			+						
873	3-methyl-octane	-				+	+	+						
884	nonane	alkane, fusel	+	+	+	+	+	+						
896	2-butylfuran	fruity, mild				+	+	+						
898	isoamyl acetate	apple, banana, ester, fresh	+	+					+	+	+	+	+	
907	nonane	alkane, fusel									+			
922	1s-()-a-pinene	fresh, herbaceous	+	+	+	+	+	+	+	+	+	+	+	+
940	5-pentanolide	-										+	+	+
962	alpha-pinene	camphore, citrus	+	+	+	+	+	+	+	+	+	+	+	+
974	beta-pinene	dry, green, hay	+	+	+	+	+	+	+	+	+	+	+	+
966	myrcene	balsamic, etheral, fruity	+	+	+	+	+	+	+	+	+	+	+	+
1033	beta-phellandrene	fruity, herbaceous	+	+	+	+	+	+	+	+	+	+	+	+
1049	limonene	citrus, fruity	+	+	+	+	+	+	+	+	+	+	+	+
1076	gamma-terpinene	citrus. etheral, fruity	+	+	+	+	+	+	+	+	+	+	+	+
1106	methylacetophenone	-				+	+	+						
1107	ethyl heptanoate	fruity	+	+	+				+	+	+	+	+	+
1135	n-nonanal	aldehydic, chlorine, citrus	+	+	+	+	+	+	+	+	+	+	+	+
1150	ethyl cyclohexanecarboxylate	-										+		
1153	2,3-diethyl-5-methylpyrazien	fragrant, hazelnut				+	+	+						
1153	e-2-nonen-1-ol	green, melon	+	+	+								+	+
1164	benzyl acetate	burnt, floral, fresh, fruity	+	+	+	+	+	+	+	+	+	+		
1187	p-methylacetophenone	almond, bitter almond, cherry	+	+	+	+	+		+	+	+	+	+	
1205	decanal	aldehydic, burnt, citrus	+	+	+				+	+	+	+	+	
1207	6-decenal	-				+	+	+						
1217	dodecane	alkane, fusel										+	+	
1248	ethyl phenylacetate	anise, cinnamon, cocoa, flaral, rose							+	+	+			
1265	2-butenoic acid, hexyl ester	fruity, green, oily, walnut												
1277	tridecane	alkane, citrus, fruity												
1300	ethyl nonanoate	fruity, rose, rum				+								
1313	nonyl acetate	fruity, leafy, sweet											+	
1272	tridecane	-									+			
1278	geranial	-	+	+										
1293	thymol	aromatic, earthy, herbaceous	+	+										
1301	tricdecane	alkane, citrus, fruity										+		
1313	butyl heptanoate	fresh, fruity, grasssy, green							+					
1316	anethole	anise, herbaceous,	+	+										
1330	nonyl acetate	fruity, leafy, sweet												+
1365	3-ethyl dodecane	-				+	+	+						
1367	eugenol	balsamic, camphore, floral								+	+			
1371	tetradecane	alkane, fusel, herbaceous	+	+	+							+	+	+
1386	n-hexyl-hexanoate	apple, fresh, fruity				+	+	+						
1369	butyl octanoate	butter, floral, fruity, green, oily	+	+	+							+	+	+
1421	propyl nonanoate	fermented, melon	+	+	+	+	+	+				+	+	+
1468	n-octylbenzene	-				+	+	+						

## Data Availability

Data is contained within the article.
